# Survey on Inter‐ and Intra‐Observer Variations of the Ultrasound Assessment of Dog Pancreases

**DOI:** 10.1111/vru.70202

**Published:** 2026-06-04

**Authors:** R. B. S. Turner, S. M. Firestone, F. R. Dunshea, C. Mansfield

**Affiliations:** ^1^ Melbourne Veterinary School, Faculty of Science University of Melbourne Werribee Victoria Australia; ^2^ School of Agriculture, Food and Ecosystem Sciences, Faculty of Sciences University of Melbourne Parkville Victoria Australia; ^3^ School of Biology, Faculty of Biological Science The University of Leeds Leeds UK; ^4^ College of Veterinary Medicine Michigan State University East Lansing Michigan USA

**Keywords:** dog, pancreas, ultrasound

## Abstract

Abdominal ultrasonography (AUS) is considered an important diagnostic tool for assessing pancreatic disease in dogs. However, AUS interpretation is highly subjective and operator‐dependent, with no prior studies evaluating observer variability in assessing the canine pancreas. This survey‐based, observer agreement study aimed to assess inter‐ and intra‐observer variability in the ultrasonographic evaluation of canine pancreases. Fifteen ultrasound images of dog pancreases were independently reviewed by 104 veterinary radiologists and radiology residents. Twenty‐four veterinary radiologists repeated the review approximately 2.5 months later. Assessments included relative echogenicity, echotexture, margination, and clinical interpretation. Agreement was quantified using Krippendorff's alpha (K*α*), prevalence‐adjusted bias‐adjusted kappa (PABAK), Cohen's kappa (κ), and intraclass correlation coefficient (ICC). Average inter‐observer agreement was poor (K*α* = 0.34), whereas intra‐observer agreement was moderate (PABAK = 0.49). Global pancreatic echogenicity and echogenicity relative to mesenteric fat had the highest inter‐observer agreement (K*α* = 0.58 and 0.53, respectively) as did echotexture assessed by a semi‐objective scale (K*α* = 0.50). However, veterinary radiologists demonstrated poor inter‐observer agreement on the clinical significance (K*α* = 0.35) and prioritized cause (K*α* = 0.14) of the pancreatic heterogeneity. Observer variables (age, training level, experience, primary practice, and accrediting organization) had minimal effect on inter‐observer agreement, apart from experience, where early‐career radiologists (<10 years’ experience) demonstrated the highest inter‐observer agreement among all groups, with moderate agreement for global echogenicity (K*α* = 0.71). These findings highlight substantial variability in the ultrasonographic interpretation of the canine pancreas, particularly in clinical decision‐making. Standardization of assessment criteria is necessary to improve consistency, clinical validity, and reduce diagnostic uncertainty.

## 1 Introduction

Abdominal ultrasonography (AUS) is a widely used, noninvasive diagnostic tool for assessing pancreatic disease in dogs [1, 2]. However, AUS is highly operator‐dependent, and its interpretation is influenced by the examiner's experience and subjective judgment. Despite this, no studies have specifically evaluated the agreement of veterinary radiologists in interpreting the canine pancreas on ultrasound.

The normal dog pancreas on ultrasound is usually described as homogeneously isoechoic to slightly hypoechoic relative to the liver and mesenteric fat, and rarely hyperechoic [2, 3, 4]. However, some studies have reported a heterogeneous echotexture, characterized by small, coalescing hyperechoic foci, as a common and normal finding in up to 40% of dogs [3]. Granger et al. reported that pancreatic echogenicity did not correlate with age, weight, or body condition score; however, increased echogenicity was associated with hyperadrenocorticism [3]. The potential relationship between these factors and echotextural heterogeneity was not investigated. Hyperechoic foci and pancreatic heterogeneity are commonly attributed to chronic pancreatitis, mineralization, fat, or fibrosis [3, 4, 5]. However, the histopathological correlation of these sonographic features is poorly defined and primarily reported in experimental models or small case series [4, 6]. This makes the interpretation of pancreatic echotextural heterogeneity challenging, particularly given that histologically confirmed chronic pancreatitis is present in 34%–65% of dogs, including many without overt clinical signs  [7, 8, 9]. Thus, in dogs presenting with gastrointestinal signs, a heterogeneous pancreas may also be attributed to normal variation, acute pancreatitis, acute‐on‐chronic pancreatitis, or chronic pancreatitis. These uncertainties raise concerns about the clinical significance of a heterogeneous pancreatic appearance and the potential for misdiagnosis or misinterpretation.

Interpretation is further complicated by the lack of standardized criteria for defining pancreatic heterogeneity. Without an accepted best practice protocol for interpretation, veterinary radiologists and veterinary clinicians may vary in their assessment, potentially leading to discrepancies in diagnoses and subsequent clinical decisions. Given that ultrasound findings can influence treatment choices, inconsistent interpretations could result in unnecessary testing, misdiagnoses, or inappropriate management strategies. Establishing clear definitions, descriptors, and reporting standards would, therefore, have both research and clinical value. Ultimately, high inter‐observer reliability is essential if pancreatic ultrasound findings are to be considered specific and clinically reliable.

This study aimed to evaluate the reliability of veterinary radiologists in interpreting canine pancreatic ultrasound images using commonly employed descriptors in clinical practice. By assessing observer variability, we sought to determine the extent of subjectivity in pancreatic ultrasound interpretation and its potential impact on clinical decision‐making.

## 2 Materials and Methods

### 2.1 Study Population

The University of Melbourne Human Research and Animal Ethics Committees granted ethical approval (ID: 2021‐22503‐22026‐3 and ID: 1613993). This prospective observer agreement study was performed in parallel to a series of investigations assessing the sonographic appearance of the dog's pancreas. From January 2016 to January 2019, abdominal ultrasound images from dogs presenting to the U‐Vet Werribee Animal Hospital (U‐Vet) were recorded. A veterinary radiology resident (R.T.) made the decision for the study's inclusion and exclusion of images. Dogs were included if the pancreas and all reference organs were adequately visualized. Exclusion criteria were ultrasonographic evidence of acute pancreatitis, pancreatic mass lesions, poor image quality, incomplete pancreatic visualization, or clinical instability precluding full examination. Dogs were scanned for a range of clinical indications and were not restricted to clinically normal animals, as the study aimed to assess observer agreement rather than define normal pancreatic appearance. Diagnosed endocrinopathies (e.g., hyperadrenocorticism and diabetes mellitus) and other comorbidities were not excluded but recorded when available. A prior history of pancreatitis was not excluded unless active ultrasonographic changes were present at the time of imaging.

### 2.2 Ultrasound Image Acquisition

**FIGURE 1:**
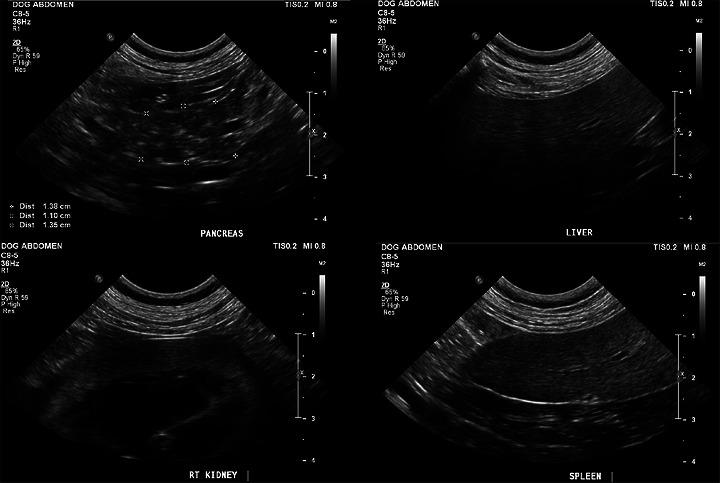
Ultrasound images of a single case showing the pancreas (top left) in relation to the liver (top right), right kidney (bottom left), and spleen (bottom right).

Image acquisition was performed by a single radiology resident (R.T.) using either the 8 MHz curvilinear or 15 MHz linear transducer (Philips EPIQ 5) and stored in DICOM format on the U‐Vet Werribee Animal Hospital picture archiving and communication system (PACS). The dogs were placed in either dorsal or lateral recumbency and scanned by either a subcostal or intercostal approach to alleviate the impediments of gastrointestinal gas and dog conformation. Once the body or right lobe of the pancreas was identified, the ultrasound image was optimized to the pancreas (depth, gain, time‐gain compensation, and the single focal point). Transverse and longitudinal images of the pancreas were performed, with the longitudinal images oriented along the pancreatic duct if visible [2]. Immediately after acquiring the pancreatic images, additional images of the adjacent right liver (RL), right kidney (RK), and spleen (S) were obtained, ensuring the ultrasound optimization remained unchanged (see Figure 1). Display of images is discussed below.

### 2.3 Data Collection and Ultrasound Image Assessment

Veterinary radiologists and residents‐in‐training programs (ACVR, ANZCVS, and ECVDI accredited) were surveyed to detail their subjective assessments of several sonographic features of the pancreas. Study data were collected and managed using REDCap electronic data capture tools hosted at The University of Melbourne [10, 11].

Participants were sought through the colleges’ list servers. Participant variables included age of participant, registered college (ACVR, ANZCVS, and ECVDI), predominant country of work, experience (years as a veterinary radiologist, year of training for residents), and primary professional role (in‐training, private practice, teleradiology, academia, or industry). Years of experience were subsequently categorized as resident, <10, 10–20, and >20 years of experience. The study utilized a convenience sample of veterinary radiologists who voluntarily participated.

On the basis of clinician time constraints, the primary investigator (R.T.) selected 15 individual dog cases (no replication) with adequate pancreatic image quality and a spectrum of sonographic features (variable thickness, echogenicity, and echotexture), independent of clinical diagnosis. No clinical information was provided to participating radiologists, and the primary investigator was excluded from image review.

For each case, raters were provided with a standardized case set comprising two static grey‐scale B‐mode  .tiff images from the same examination: (1) a single representative image of the pancreas in isolation and (2) a composite image displaying the pancreas alongside all reference organs in a standardized layout for comparison (see Figure 1). Participants could use digital zoom, but  .tiff format was used to limit post‐acquisition windowing or leveling adjustments. No cine loops were provided. Each case was assessed using 13 predefined subjective ultrasonographic descriptors (Table 1). Minimal descriptor definitions were provided to allow independent interpretation on the basis of commonly used semi‐qualitative ultrasonographic terminology, with the intention of assessing natural variability in descriptor application within the veterinary radiologist population [12]. Global echogenicity was intended to capture the reviewer's overall first visual impression of pancreatic brightness before structured relative comparison. This reflects common clinical reporting behavior, where an initial gestalt assessment precedes formal reference‐based evaluation. A continuous 0–100 sliding scale was selected to permit greater granularity and allow interval‐level statistical analysis (ICC), rather than restricting assessment to coarse ordinal categories.

**TABLE 1:** List of ultrasound and clinical descriptors of the pancreas.

Category	Descriptor
*Ultrasound categories*	*Ultrasound categories*
Global echogenicity	First impression of pancreas is echogenicity without relative reference and categorized as hyperechoic, isoechoic, or hypoechoic
Relative echogenicity	Hyperechoic, isoechoic, or hypoechoic relative to: mesentery (M), right liver (RL), right renal cortex (RK), and spleen (S)
Uniformity of echogenicity and echotexture	Homogeneous or heterogeneous
Type of echotexture	Fine echotexture or coarse echotexture
Degree of heterogeneity (heterogeneity scale)	Subjective sliding scale (0–100) from minimally heterogeneous (homogeneous) to markedly heterogeneous
Distribution of heterogeneity	Diffuse, focal, or multifocal
Location of heterogeneity	Generalized through parenchyma, periductal, or peripheral
Visibility of pancreatic margins	Present or absent
Appearance of pancreatic margins^a^	Ill‐defined, smooth, undulating, or nodular
*Clinical categories*	
Sonographically normal or abnormal	Normal or abnormal
What would you list as the cause(s) of the heterogeneity? (Choose as many as required)	Normal variation (but unknown cause); chronic pancreatitis; acute or acute‐on‐chronic pancreatitis; fibrosis; fat infiltration; dystrophic mineralization; neoplasia; and others
What would you list as the primary cause of the heterogeneity? (choose one)	Normal variation (but unknown cause); chronic pancreatitis; acute or acute‐on‐chronic pancreatitis; fibrosis; fat infiltration; dystrophic mineralization; neoplasia; and other (record option)
Clinical significance	(I) Normal; (II) incidental or clinically insignificant; (III) possibly clinically significant but correlated with clinical findings; (IV) likely clinically significant, and diagnostic or treatment intervention may be required; (V) or definitely clinically significant, and diagnostic or treatment intervention is required. The five categories were collapsed into three broader categories to facilitate interpretation: (1) normal/incidental (I + II), (2) possibly clinical (III), and (3) likely or definitively clinical (IV + V)

^a^ Calipers delineating the margins of the pancreas and, where identifiable, the pancreatic duct were included on the provided static image. Cine loops and Doppler ultrasound images were not available for review.

The first survey (inter‐observer assessment) was open for 2 months. A second survey assessing intra‐observer agreement, and using the exact cases used in the first survey, was released 1 month later and remained open for 3 months.

### 2.4 Data Analyses

Prior to the study, the minimum required sample size for the survey was calculated to be 88–278 veterinary radiologists. This estimate was based on an assumed population of 1000 veterinary radiologists, a margin of error of ±5%–10%, and a 95% confidence interval (CI). The population size was approximated from the number of members listed on the ACVR, ANZCVS, and ECVDI websites.

Inter‐observer reliability among veterinary radiologists assessing 13 sonographic features of 15 canine pancreases was evaluated using Krippendorff's alpha (K*α*). This method was selected for its ability to accommodate multiple raters, data type (ordinal, nominal, and interval data), and potentially missing values [13]. Incomplete survey responses were retained in the analysis because K*α* calculates agreement using all available pairwise observations and does not require complete datasets. The survey was structured so that all responses within a case set had to be completed before proceeding to the next case; therefore, missing data occurred sequentially as a result of survey attrition rather than selective omission of particular variables. K*α* was chosen in part to appropriately account for this attrition and to minimize potential bias associated with a reduced sample size. The ratings were analyzed using the web‐based K*α* calculator [14], which computed reliability coefficients reflecting the level of agreement beyond chance. The interpretation of K*α* values followed established guidelines: Negative values indicate systematic disagreement, 0 indicates no agreement, <0.67 suggests poor agreement, 0.67–0.79 indicates moderate agreement, ≥0.80 represents strong agreement, and 1.0 signifies perfect agreement [14].

Intra‐observer reliability was assessed in a subset of veterinary radiologists who completed the survey a second time, as repeat participation was voluntary and required opt‐in at the conclusion of the first survey. For nominal data, non‐weighted Cohen's kappa and the prevalence‐adjusted bias‐adjusted kappa (PABAK) were calculated. For ordinal data, weighted Cohen's kappa and PABAK were used to account for potential effects of prevalence and bias. PABAK values were selected for reporting due to their greater stability in the presence of skewed category distributions and are presented in the final tables. For continuous data, intraclass correlation coefficients (ICC) were estimated using a two‐way random‐effects model with a single‐rater unit and an absolute agreement definition. Analyses were performed using Jamovi and the Seolmatrix module (version 2.6). Kappa (κ) and PABAK values were interpreted on the basis of Landis and Koch's guidelines: Negative values indicate systematic disagreement, 0 indicates no agreement, ≤0.20 suggests slight agreement, 0.21–0.40 fair agreement, 0.41–0.60 moderate agreement, 0.61–0.80 substantial agreement, and 0.81–1.00 almost perfect agreement. The interpretation of intraclass correlation coefficient values followed established guidelines: <0.50 suggests poor reliability, 0.50–0.75 moderate reliability, 0.75–0.9 good reliability, and >0.9 excellent reliability [15]. To enhance robustness for both the inter‐ and intra‐observer reliability, bootstrapping with 1000 resamples was applied to estimate 95% CIs [16].

The effect of observer variables (age, experience, training level, accrediting organization, and primary practical role) on inter‐observer agreement of pancreatic global echogenicity and heterogeneity was also assessed by stratifying the agreement for the observer characteristics.

## 3 Results

### 3.1 Patient Data

The 15 selected cases comprised 7 female‐spayed, 6 male‐neutered, and 2 female entire dogs, with a median age of 10.6 years (range 5.4–14 years). Body weights ranged from 4.9 to 29.2 kg, and body condition scores from 3 to 9/9. One dog had diabetes mellitus, and two had hyperadrenocorticism; the remaining cases were investigated for conditions unrelated to primary pancreatic disease.

### 3.2 Survey Response Data

The first survey had 131 responses. Eighteen surveys were removed from the analysis because of no ultrasound data entry, and nine were removed due to duplication. Of the 104 surveys analyzed, 38 had complete data entry (37% completion). The second survey had 24 complete responses (100%). The median time between studies was 2.6 months (range: 1.5–4.1 months).

### 3.3 Demographic Data

Of the 104 respondents, 80 (77%) participants were board‐certified veterinary radiologists, 20 (19%) were residents‐in‐training, and 4 (4%) were residency‐trained (registrar). Of the 80 board‐certified veterinary radiologists, 58 (73%) were diplomats of the ACVR, 15 (19%) were diplomats of the ECVDI, 5 (6%) were fellows of the ANZCVS, and 2 (2%) were diplomats of both the ACVR and ECVDI. Of the 104 respondents, 47 (45%) predominantly practiced in a private referral hospital, 35 (34%) in academia, and 22 (21%) in teleradiology. Of the 24 residents and registrars, 19 were in academic institutes, and 5 were in private referral hospitals. Of all the respondents, 67 (64%) practiced in North America, 16 (15%) in Europe, 14 (14%) in Australasia, and 7 (7%) in the United Kingdom. The board‐certified veterinary radiologists had a median of 10.5 years of radiology experience (range: 0–45 years) and a median age of 31–40 years (47% of the group).

### 3.4 Reliability

A summary of pancreatic characteristics and the inter‐ and intra‐observer variability for each descriptor is shown in Table 2. Veterinary radiologists showed poor inter‐observer (K*α* = 0.34) and moderate intra‐observer (PABAK = 0.49) agreement in assessing the ultrasonographic and clinical features of the dog's pancreases. The metric with the highest level of agreement was the assessment of the pancreas's global echogenicity (inter‐observer K*α* = 0.58 [95% CI: 0.34, 0.74]; intra‐observer PABAK = 0.67 [95% CI: 0.58, 0.75]), relative echogenicity to mesenteric fat (inter‐observer K*α* = 0.53 [95% CI: 0.29, 0.70]; intra‐observer PABAK = 0.60 [95% CI: 0.51, 0.68]), and the semi‐quantitative scaled assessment of heterogeneity (inter‐observer K*α* = 0.50 [95% CI: 0.30, 0.59]; intra‐observer ICC = 0.72 [95% CI: 0.66, 0.78]).

**TABLE 2:** Inter‐ and intra‐observer agreement for ultrasound variables of the pancreas.

		Inter‐observer agreement	Inter‐observer agreement	Intra‐observer agreement (*n* = 24)	Intra‐observer agreement (*n* = 24)
Category		K*α* [95% CI]	Responses^a^ (*n*/1560)	Cohen's *κ* [95% CI]	PABAK [95% CI]
Average agreement		0.34		0.52	0.49
*Ultrasound categories*	*Ultrasound categories*	*Ultrasound categories*	*Ultrasound categories*	*Ultrasound categories*	
Global echogenicity		0.58 [0.34, 0.74]	786	0.68 [0.57, 0.78]	0.67 [0.58, 0.75]
Relative echogenicity	Mesenteric fat	0.53 [0.29, 0.70]	748	0.63 [0.55, 0.72]	0.60 [0.51, 0.68]
Relative echogenicity	Liver	0.40 [0.20, 0.55]	749	0.45 [0.38, 0.53]	0.32 [0.22, 0.42]
Relative echogenicity	Right RENAL CORTEX	0.32 [0.11, 0.53]	748	0.49 [0.41, 0.57]	0.36 [0.26, 0.46]
Relative echogenicity	Spleen	0.38 [0.17, 0.58]	748	0.55 [0.47, 0.63]	0.49 [0.39, 0.57]
Uniformity of echotexture	Homogeneous or heterogeneous	0.45 [0.20, 0.60]	786	0.56 [0.45, 0.66]	0.57 [0.47, 0.65]
Type of echotexture	Fine or coarse	0.35 [0.14, 0.51]	786	0.53 [0.42, 0.63]	0.53 [0.43, 0.61]
Heterogeneity scale		0.50 [0.30, 0.59]	786	0.72 [0.66, 0.78]^β^	
Distribution of echotexture	Diffuse, focal, and multifocal	0.16 [0.08, 0.22]	783	0.38 [0.30, 0.46]	0.46 [0.36, 0.55]
Location of heterogeneity	Generalized, periductal, and peripheral	0.20 [0.08, 0.29]	733	0.41 [0.32, 0.51]	0.54 [0.44, 0.63]
Visibility of margins	Yes or no	0.25 [0.07, 0.34]	784	0.61 [0.51, 0.72]	0.78 [0.71, 0.84]
Appearance of pancreatic margins^c^	Ill‐defined, smooth, undulating, and nodular	0.20 [0.13, 0.27]	784	0.43 [0.36, 0.50]	0.39 [0.29, 0.49]
*Clinical categories*	*Clinical categories*	*Clinical categories*	*Clinical categories*	*Clinical categories*	
Sonographically normal or abnormal		0.33 [0.14, 0.49]	784	0.57 [0.47, 0.68]	0.58 [0.49, 0.66]
Primary cause of heterogeneity		0.14 [0.07, 0.20]	782	0.42 [0.36, 0.48]	0.25 [0.14, 0.35]
Clinical significance		0.35 [0.14, 0.52]	782	0.47 [0.41, 0.54]	0.27 [0.17, 0.37]
Aggregated clinical significance^d^		0.26 [0.11, 0.39]	782	0.53 [0.45, 0.61]	0.52 [0.43, 0.61]

*Note*: K*α*, Krippendorf's alpha; *κ*, kappa (Cohen's or PABAK).

Abbreviation: PABAK, prevalence‐adjusted bias‐adjusted kappa.

^a^ The maximum number of responses for each variable, if all 104 raters had rated all 13 items, was 1560.

^b^ Continuous interval and agreement assessed with the intraclass correlation coefficient (ICC).

^c^ Calipers delineating the margins of the pancreas and, where identifiable, the pancreatic duct were included on the provided static image. Cine loops and Doppler ultrasound images were not available for review.

^d^ The five original categories of clinical significance were collapsed into three broader categories to facilitate interpretation: (1) normal/incidental, (2) possibly clinical, and (3) likely or definitively clinical.

Of the 784 responses, 51% (397/784) of pancreases were considered sonographically normal and 49% (387/784) abnormal, with poor inter‐observer agreement and moderate intra‐observer agreement (see Table 2).

When considering the primary cause of the pancreatic echotexture (782 responses), there was poor inter‐observer and fair intra‐observer agreement, with 50% (389/782) of the observers considered these pancreases normal, 15% (119/782) categorized the primary cause of the heterogeneity as chronic pancreatitis, 13% (102/782) acute or acute‐on‐chronic pancreatitis, 9% (71/782) fibrosis, 8% (59/782) fat infiltration, and the remaining 5% (42/782) due to other causes (e.g., dystrophic mineralization, neoplasia, or other).

Regarding clinical significance (782 responses), there was poor inter‐observer and fair‐to‐moderate intra‐observer agreement. Most responses classified findings as clinically normal (39%, 306/782) or incidental/clinically insignificant (21%, 164/782). A further 31% (242/782) considered the findings possibly clinically significant, requiring clinical correlation; 8% (62/782) as likely clinically significant, with potential need for diagnostic or therapeutic intervention; and 1% (8/782) as definitely clinically significant, requiring intervention.

Veterinary radiologist characteristics appeared to have some influence on inter‐observer agreement for pancreatic global echogenicity and the pancreatic heterogeneity scale. This is summarized in Table 3. Notably, early‐career radiologists (10 years’ experience) demonstrated the highest agreement among all groups, with moderate inter‐observer agreement observed for global echogenicity (K*α* = 0.71 [95% CI: 0.46, 0.88]).

**TABLE 3:** Inter‐observer agreement for global echogenicity, pancreatic heterogeneity, and clinical interpretation stratified by veterinary radiologist characteristics.

		Inter‐observer agreement K*α* [95% CI]	Inter‐observer agreement K*α* [95% CI]	Inter‐observer agreement K*α* [95% CI]	Inter‐observer agreement K*α* [95% CI]	Inter‐observer agreement K*α* [95% CI]
Veterinary radiologist characteristics	Number of veterinary radiologists (*n*/24)	Global echogenicity	Heterogeneity scale	Sonographically normal or abnormal	Primary cause of heterogeneity	Clinical significance
Age (years)						
21–30	9	0.26 [−0.28, 0.62]	0.60 [−0.07, 0.78]	0.26 [−0.23, 0.59]	−0.01 [−0.24, 0.16]	0.24 [−0.14, 0.47]
31–40	49	0.64 [0.38, 0.80]	0.51 [0.34, 0.60]	0.32 [0.10, 0.51]	0.11 [0.05, 0.16]	0.22 [0.08, 0.34]
41–50	23	0.62 [0.32, 0.82]	0.62 [0.37, 0.76]	0.49 [0.19, 0.70]	0.19 [0.07, 0.29]	0.27 [0.11, 0.39]
51–60	13	0.51 [0.19, 0.74]	0.35 [0.06, 0.56]	0.32 [0.08, 0.52]	0.25 [0.12, 0.35]	0.23 [0.08, 0.34]
60+	10	0.42 [0.13, 0.66]	0.56 [0.34, 0.69]	0.20 [−0.03, 0.39]	0.14 [0.01, 0.24]	0.06 [−0.04, 0.16]
Years of experience						
Resident	24	0.54 [0.25, 0.75]	0.53 [0.26, 0.65]	0.32 [0.11, 0.49]	0.14 [0.07, 0.21]	0.21 [0.05, 0.36]
<10 years	34	0.71 [0.46, 0.88]	0.48 [0.29, 0.59]	0.31 [0.08, 0.48]	0.09 [0.02, 0.14]	0.18 [0.06, 0.27]
10–20 years	28	0.56 [0.27, 0.74]	0.55 [0.31, 0.67]	0.43 [0.17, 0.64]	0.18 [0.07, 0.26]	0.29 [0.13, 0.41]
>20 years	18	0.49 [0.24, 0.71]	0.47 [0.25, 0.61]	0.23 [0.04, 0.41]	0.13 [0.04, 0.20]	0.09 [0.01, 0.15]
Training level						
Consultant	80	0.59 [0.344, 0.75]	0.50 [0.31, 0.61]	0.33 [0.13, 0.51]	0.14 [0.07, 0.20]	0.18 [0.08, 0.27]
Resident trained	4	0.40 [−0.01, 0.76]	0.61 [0.29, 0.80]	0.18 [−0.17, 0.48]	0.11 [−0.05, 0.24]	0.11 [−0.11, 0.36]
Resident‐in‐training	20	0.63 [0.32, 0.82]	0.50 [0.18, 0.63]	0.29 [0.09, 0.46]	0.13 [0.03, 0.21]	0.28 [0.08, 0.44]
Primary practice						
Private practice	47	0.66 [0.43, 0.82]	0.50 [0.30, 0.62]	0.32 [0.12, 0.48]	0.13 [0.06, 0.18]	0.19 [0.08, 0.27]
Teleradiology	22	0.58 [0.29, 0.77]	0.52 [0.30, 0.65]	0.38 [0.09, 0.61]	0.16 [0.05, 0.23]	0.20 [0.07, 0.30]
Academia	35	0.48 [0.20, 0.69]	0.50 [0.27, 0.62]	0.33 [0.11, 0.50]	0.15 [0.07, 0.22]	0.20 [0.07, 0.30]
Accredited organization						
ACVR	71	0.60 [0.37, 0.75]	0.49 [0.28, 0.59]	0.37 [0.15, 0.54]	0.16 [0.08, 0.23]	0.20 [0.09, 0.28]
ANZCVS	8	0.60 [0.29, 0.84]	0.56 [0.31, 0.70]	0.29 [0.06, 0.51]	0.17 [0.05, 0.29]	0.17 [0.03, 0.28]
ECVDI	25	0.50 [0.14, 0.70]	0.47 [0.26, 0.57]	0.24 [0.02, 0.40]	0.09 [0.00, 0.14]	0.13 [0.00, 0.25]

*Note*: K*α* Krippendorf's alpha.

## 4 Discussion

This study highlights substantial variability among veterinary radiologists in the interpretation of canine pancreatic sonographic features and the subsequent clinical significance assigned to these findings. This limited reliability mirrors challenges reported in the human medical literature, where attempts to characterize and clinically apply sonographic features of the pancreas have proven difficult [17, 18, 19, 20].

Prior to this investigation, inter‐observer agreement regarding pancreatic heterogeneity and echogenicity in dogs had not been formally assessed. Granger et al. previously described the distribution of echogenicity patterns in clinically normal dogs but did not evaluate inter‐observer reliability or correlate subjective assessments with objective, quantitative measures [3]. Our study addresses this gap and demonstrates that agreement is generally poor. Additionally, this study also highlights the reliance on peripancreatic fat when assessing relative pancreatic echogenicity, which showed a similarly poor, yet the relatively highest, inter‐observer agreement among the evaluated features.

Comparisons with human medicine reveal that observer agreement for pancreatic sonographic features is also imperfect, with moderate mean inter‐observer agreement (mean *κ* = 0.46) and improved intra‐observer agreement (mean *κ* = 0.66) [17, 18, 19, 20, 21, 22, 23, 24, 25, 26, 27, 28]. Notably, the veterinary radiologists in our study exhibited lower overall inter‐observer agreement (K*α* = 0.34) than reported for medical endosonographers. However, when focusing specifically on features, such as heterogeneity and hyperechoic foci, the level of agreement was like that reported in human studies (*κ* = 0.38–0.50). Studies involving larger numbers of observers and lacking consensus or training protocols in human medicine also reported similarly poor‐to‐modest agreement, suggesting that variability is an inherent challenge across disciplines [23, 27].

Operator experience is frequently cited as a factor contributing to interpretive variability. Some studies have shown that specialized training and simplified assessment criteria can improve inter‐observer agreement, whereas others have found no significant effect of experience or training once a certain threshold is reached [19, 20, 28]. In this study, early‐career radiologists (<10 years’ experience) demonstrated the highest inter‐observer agreement for global echogenicity (K*α* = 0.71). However, as CIs overlapped across experience groups, this observation should be considered exploratory and interpreted cautiously. Overall, the findings suggest that although experience may play a role, the complexity and subjectivity of sonographic interpretation are persistent obstacles.

The underlying causes of sonographic features, such as hyperechoic foci and pancreatic heterogeneity in dogs, are not well understood. These findings may reflect a spectrum of conditions, including normal anatomical variation, chronic pancreatitis, fibrosis, nodular hyperplasia, and steatosis, with chronic pancreatitis often considered the most clinically relevant [3]. Our data underscore the uncertainty in classifying pancreatic changes as normal or pathologic, as well as the lack of consensus regarding the etiology of observed heterogeneity. This diagnostic inconsistency is concerning, given the routine use of ultrasound in dogs with gastrointestinal signs and the high prevalence of histologic chronic pancreatitis in the canine population, often without clinical correlation [7].

Diagnosing chronic pancreatitis remains particularly challenging, especially with noninvasive modalities such as ultrasound. Although ultrasound is theoretically valuable, our results demonstrate poor reliability in both the identification and interpretation of relevant sonographic features. For example, Granger et al. found that 40% of clinically normal dogs exhibited heterogeneous pancreatic echotexture, raising questions about the specificity of this finding [3]. Unlike human medicine, where standardized frameworks, such as the Japanese or Rosemont criteria, are used to improve diagnostic consistency, veterinary radiology lacks such validated systems [19, 22]. Even with structured criteria, diagnostic accuracy ultimately requires correlation with clinical signs and histopathology—an important consideration given the high prevalence of subclinical chronic pancreatitis in dogs [7, 17].

Several methodological limitations may have influenced our results. The intentional selection of cases demonstrating a spectrum of sonographic appearances may have enriched heterogeneity and does not fully represent routine case‐mix variability in general practice settings. Although respondents were predominantly from North America and Europe, other regions, including Africa, Asia, and Latin America, were underrepresented. Differences in regional training pathways, professional structures, and reporting practices may, therefore, limit the generalizability of these findings beyond the surveyed population. Future studies with broader geographic representation would help determine whether these findings are consistent across different professional and training environments. The response rate from veterinary radiologists was acceptable for the initial survey but declined substantially for the follow‐up, likely due to survey length and repetitive structure, which may have contributed to fatigue and survey attrition. The reduced sample size at follow‐up affected precision, although the use of K*α*, the expertise of respondents, and relative consistency with the human literature suggest our findings are representative of the broader veterinary radiologist community [17].

Technical factors inherent to ultrasound imaging, such as probe frequency, acoustic coupling, patient size, image acquisition protocols, image format (e.g., DICOM), and absence of cine loops, may have contributed to variability in interpretation [28]. The lack of dynamic scanning likely disproportionately affected descriptors requiring spatial and margin assessment, including distribution of echotexture (K*α* = 0.16), location of heterogeneity (K*α* = 0.20), and visibility and appearance of the pancreatic margins (K*α* = 0.20–0.25). These features may benefit from real‐time interrogation across multiple planes. Because all reviewers evaluated identical static images with fixed acquisition parameters and visible reference organs, variability in global echogenicity reflects interpretive differences rather than machine‐dependent gain variation. The absence of standardized operational definitions for certain descriptors (e.g., undulating vs. nodular margins) may have further contributed to variability, reflecting differences in individual interpretive thresholds. However, the primary focus was on veterinary radiologists’ interpretation of the static grey‐scale images, rather than image acquisition. Future studies should assess repeatability using different ultrasound machines and operators and consider the potential role of radiomics in providing more objective and quantitative assessments. However, the adoption of such approaches will require collaborative efforts and standardization of image acquisition and analysis protocols, which are not yet established in veterinary ultrasound examinations [29].

Improving rater reliability may be achieved by developing and implementing more specific, yet straightforward, criteria for pancreatic sonographic evaluation [17, 19, 22]. Training based on these consensus‐based criteria could enhance consistency, allowing for more accurate correlation between sonographic findings and clinical outcomes, thereby improving both clinical practice and research applications.

This study reveals that veterinary radiologists have poor agreement when interpreting canine pancreatic ultrasound images, highlighting the subjective nature and lack of standardization in current practice. Recognizing this variability is a crucial first step toward developing consensus‐driven, objective criteria for pancreatic evaluation. By addressing these inconsistencies, future research can pave the way for more reliable diagnoses and better clinical outcomes for canine patients.

## Author Contributions

*Conception and design*: R. B. S. Turner, S. M. Firestone, and C. Mansfield. *Acquisition of data*: R. B. S. Turner. *Analysis and interpretation of data*: R. B. S. Turner and S. M. Firestone. *Drafting the article*: R. B. S. Turner. *Revising article for intellectual content*: R. B. S. Turner, S. M. Firestone, F. Dunshea, and C. Mansfield. *Final approval of the completed article*: R. B. S. Turner, S. M. Firestone, F. Dunshea, and C. Mansfield.

## Disclosure

GRRAS checklist completed.

## Conflicts of Interest

The authors declare no conflicts of interest.

## Data Availability

The datasets used and/or analyzed during the current study are available from the corresponding author on reasonable request.
